# LexMAL: A quick and reliable lexical test for Malay speakers

**DOI:** 10.3758/s13428-023-02202-5

**Published:** 2023-09-01

**Authors:** Soon Tat Lee, Walter J. B. van Heuven, Jessica M. Price, Christine Xiang Ru Leong

**Affiliations:** 1 School of Psychology, University of Nottingham Malaysia, Jalan Broga, 43500 Semenyih, Selangor Malaysia; 2 University of Nottingham, Nottingham, UK

**Keywords:** Language proficiency, Malay, Vocabulary test, Bilingualism

## Abstract

Objective language proficiency measures have been found to provide better and more consistent estimates of bilinguals’ language processing than self-rated proficiency (e.g., Tomoschuk et al., 2019; Wen & van Heuven, 2017a). However, objectively measuring language proficiency is often not possible because of a lack of quick and freely available language proficiency tests (Park et al., 2022). Therefore, quick valid vocabulary tests, such as LexTALE (Lemhöfer & Broersma, 2012) and its extensions (e.g., LexITA: Amenta et al., 2020; LEXTALE-FR: Brysbaert, 2013; LexPT: Zhou & Li, 2022) have been developed to reliably assess language proficiency of speakers of various languages. The present study introduces a Lexical Test for Malay Speakers (LexMAL), which estimates language proficiency for Malay first language (L1) and second language (L2) speakers. An initial 180-item LexMAL prototype was evaluated using 60 Malay L1 and 60 L2 speakers in Experiment 1. Sixty words and 30 nonwords with the highest discriminative power that span across the full difficulty range were selected for the final LexMAL based on point-biserial correlations and an item response theory analysis. The validity of LexMAL was demonstrated through a reliable discrimination between L1 and L2 speakers, significant correlations between LexMAL scores and performance on other Malay language tasks (i.e., translation accuracy and cloze test scores), and LexMAL outperforming self-rated proficiency. A validation study (Experiment 2) with the 90-item final LexMAL tested with a different group of Malay L1 (*N* = 61) and L2 speakers (*N* = 61) replicated the findings of Experiment 1. LexMAL is freely available for researchers at www.lexmal.org.

## Introduction

The language proficiency of bilinguals affects the representations and processing of the languages that they speak (see Jiang, 2015 for a review). Therefore, it is important for experimental studies to measure language proficiency in first language (L1) (Brysbaert et al., 2016; Hulstijn, 2015; Lee et al., 2022) and second language (L2) speakers (Diependaele et al., 2013; Wen & van Heuven, 2017a; Zhang et al., 2020). Objective language measures such as vocabulary size tests have been shown to provide reliable and accurate estimation of individual differences of language proficiency among bilinguals (e.g., Lemhöfer & Broersma, 2012; Tomoschuk et al., 2019; Zhang et al., 2020). However, systematic reviews (Park et al., 2022; Surrain & Luk, 2019) show that objective language proficiency measures are not consistently used whenever language proficiency is measured, with less than 50% of bilingual research from the last decade using an objective language proficiency measure to assess participants' language proficiency.

One of the reasons that researchers do not use objective language proficiency measures is that such tests are not freely available for all languages (Park et al., 2022). Furthermore, standardized language proficiency tests might involve costs (e.g., International English Language Testing System, IELTS) or they take a long time to administer (e.g., 40 min for the Vocabulary Size Test, Nation & Beglar, 2007). In addition, objective language proficiency measures might not be available in understudied languages. For instance, there is currently no freely available quick Malay proficiency test, although there are 377 million Malay speakers in the world.

The Malay language belongs to the Austronesian family that is commonly spoken in Southeast Asian countries such as Malaysia, Brunei, Indonesia, and Singapore (Lee et al., 1998; Lee & Wheldall, 2011; Tan et al., 2009). Psycholinguistic studies conducted in Malaysia often use Malay for cross-linguistic comparisons with English because Malaysia has a rather unique population of bilingual English speakers (e.g., Rahman et al., 2018; Rusli & Montgomery, 2020). Many Malaysians can be considered as early Malay–English bilinguals because both languages are acquired before starting school (Jin et al., 2013). Furthermore, both Malay and English are commonly used in multiple daily contexts from early childhood. This makes the Malaysian population highly proficient in both languages and therefore rather interesting for research on bilingualism. Because many Malaysians acquire Malay and English from a very early age, it is difficult to assess Malay proficiency just based on the order of acquisition or exposure. Therefore, a quick objective test of Malay proficiency would be very useful for this population.

Studies that involved Malay-speaking bilinguals have so far either assumed “native-like” proficiency of Malay L1 speakers (e.g., Lee & Low, 2014; Yap et al., 2017), or used self-ratings to estimate the speakers’ language proficiency (e.g., Jalil et al., 2011; Rahman et al., 2018; Rusli & Montgomery, 2020). However, the assumption of “native-like” proficiency is not always reliable because the language proficiency of L1 speakers (e.g., vocabulary size) could vary substantially according to the speakers’ language experience (e.g., whether a person reads) (Brysbaert et al., 2016; Hulstijn, 2015). Furthermore, language proficiency estimated by self-ratings can be affected by individual and group differences (Tomoschuk et al., 2019). For instance, L1 speakers may compare their proficiency with other L1 speakers, whereas second language (L2) speakers might refer to the best L2 speaker model they have in mind. Such individual differences in the choice of a proficiency reference could result in unreliable ratings, especially for a heterogenous group (e.g., relatively “noisy” group of participants with a broad range of language proficiency) (Brysbaert, 2013; Chan & Chang, 2018). In addition, participants of different language combinations (e.g., Spanish–English, Chinese–English) or language background (e.g., heritage speakers or recently immigrated bilinguals) have been found to vary in their accuracy of self-rated proficiency, rendering difficulty in comparing self-rated proficiency across different participant groups (Lemhöfer & Broersma, 2012; Tomoschuk et al., 2019). Assuming “native-like” proficiency or using self-ratings to measure language proficiency, therefore, may not always be reliable and valid as a language proficiency estimate in bilingual research (Cheng et al., 2021; Li & Zhang, 2021; Tomoschuk et al., 2019). Hence, there is a need for a valid language proficiency test that could reliably quantify the language proficiency of Malay speakers with different proficiency levels.

Vocabulary tests have been used as an objective language proficiency measure because vocabulary knowledge is believed to be central to language competence (Beglar & Nation, 2013; Braze et al., 2007; Nguyen & Nation, 2011; Meara, 1996; Perfetti & Hart, 2002). Researchers have used different definitions for the measurement of vocabulary knowledge in view of its multifaceted unidimensional construct (González-Fernández & Schmitt, 2020; Laufer & Goldstein, 2004; Webb, 2013). In general, vocabulary knowledge can be measured by using two different vocabulary knowledge components: depth and breadth (Anderson & Freebody, 1981; Schmitt, 2014; Webb, 2013). Depth of vocabulary knowledge refers to the quality of vocabulary knowledge. It is conceptualized as the overall degree of knowledge of all the word knowledge aspects involved (e.g., knowledge of collocation: how words should be used together, and word association: how words can be used interchangeably) (Nation, 2013). However, there is currently no consensus on how it can be accurately measured in view of the multifaceted and interrelated nature of the vocabulary components (González-Fernández & Schmitt, 2020; Schmitt, 2014; Webb, 2013).

Because of the complexity of measuring vocabulary depth, most vocabulary tests designed for research have focused on the breadth of vocabulary knowledge, or the number of words known by a person (Schmitt, 2014). The score of a typical vocabulary size test can be used to estimate performance in language tasks. For instance, Nation (2006) showed that at least 8000 word families are needed for language learners to perform various language tasks fluently (e.g., reading newspapers, watching movies). Furthermore, vocabulary size has a strong correlation with various aspects of word knowledge (e.g., collocations, multiple meanings) (González-Fernández & Schmitt, 2020) and word processing (e.g., listening comprehension) (Andringa et al., 2012; Rodríguez-Aranda & Jakobsen, 2011; Yap et al., 2012). These findings support the use of a vocabulary size test as a language proficiency estimate in bilingual research to account for individual differences in terms of language proficiency or ability.

In the field of psycholinguistics, the Lexical Test for Advanced Learners of English (LexTALE) developed by Lemhöfer and Broersma (2012) has been widely used to measure the English proficiency of advanced learners of English. This unspeeded yes/no vocabulary test is short and time efficient, containing a total of 60 items (40 words and 20 nonwords) with the ratio of words and nonwords being 2:1. Test-takers are required to indicate if letter strings are existing English words by responding “yes” or “no”. LexTALE is freely available in the form of paper-and-pencil and online formats. Previous studies have demonstrated the validity of LexTALE by showing its ability to explain language performance measured by other language tasks such as lexical decision and visual word recognition tasks (e.g., Diependaele et al., 2013; Lemhöfer & Broersma, 2012; Wen & van Heuven, 2017b). Objective language proficiency measures like LexTALE are encouraged to be used as standard language proficiency measures in bilingual research to promote generalization and accumulation of research findings across studies (Diependaele et al., 2013; Lemhöfer & Broersma, 2012; Zhang et al., 2020).

LexTALE has its Dutch and German equivalent versions, designed with their difficulty level being matched as closely as possible, to allow cross-linguistic comparisons (Lemhöfer & Broersma, 2012). Inspired by the English LexTALE and its extensions to Dutch and German, other researchers have developed similar lexical tests for estimating language proficiency in other languages. To date, lextale extensions are available for French (LEXTALE-FR: Brysbaert, 2013), Spanish (Lextale-Esp: Izura et al., 2014), Chinese (LEXTALE_CH: Chan & Chang, 2018; LexCHI: Wen et al., 2023), Italian (LexITA: Amenta et al., 2020), Portuguese (LextPT: Zhou & Li, 2022) and Finnish (Lexize: Salmela et al., 2021). These lextale extensions were not matched against LexTALE in terms of the word stimuli and the items’ difficulty level. Instead, they were designed to measure vocabulary size of speakers from a wider language proficiency range (i.e., L1 and L2 speakers). In these tests, more items were included, and overall difficulty level was increased to improve their reliability and suitability to measure language proficiency of both L1 and L2 speakers (Amenta et al., 2020; Brysbaert, 2013; Chan & Chang, 2018; Izura et al., 2014).

To be able to discriminate between the vocabulary size of test-takers with different proficiency levels, the lextale extensions included a good blend of high- and low-frequency words selected from subtitle-based lexical database (e.g., SUBTLEX-ESP, Cuetos et al., 2011). L1 speakers who are highly proficient in the target language are expected to have acquired knowledge of most high-frequency words, whereas their knowledge of low-frequency lexical items varies depending on their language experience (Hulstijn, 2015). Less-proficient L2 speakers, on the other hand, may show relatively greater variation even in knowledge of high-frequency words. Therefore, these lextale extensions have been shown to successfully discriminate between the vocabulary size of L1 and L2 speakers with large effect sizes, *d*s ≥ 2.52.

To address the need of a reliable and valid quick Malay proficiency measure, we followed the standard procedures from previous lextale extensions (Amenta et al., 2020; Brysbaert, 2013; Chan & Chang, 2018; Izura et al., 2014; Salmela et al., 2021; Wen et al., 2023; Zhou & Li, 2022) to develop a lexical test for estimating language proficiency in Malay (LexMAL). The LexMAL prototype included a good mix of high- and low-frequency words to ensure that the test can distinguish between the vocabulary size of Malay L1 and L2 speakers using the same scale^1^. In contrast to previous lextale extensions, the item selection for LexMAL was based on the frequency of occurrences taken from the Malay Lexicon Project (Yap et al., 2010), a lexicon corpus based on daily newspapers published in Malaysia. Because Malay-speaking bilinguals in Malaysia are proficient in daily conversation using Malay, vocabulary sampled from written materials offers a more diverse range of rare words that are not limited to daily conversational topics to assess and discriminate their vocabulary knowledge (e.g., “*salasilah*/genealogy”, “*kerawang*/fretwork”, and “*kendur*/loose”). In other words, the word items in LexMAL are likely to reflect vocabulary items used in standard Malay (e.g., Malay used in formal writing) rather than spoken colloquial variations (e.g., Malay used in informal daily conversation). For example, word items in LexMAL do not test word knowledge of colloquial word forms that are used in daily conversation such as “*(de)kat*/at”, “*okey*/okay” and “*kaukau*/thick”.

Following previous studies (e.g., Wen et al., 2023), two experiments were conducted to construct and validate LexMAL. Experiment 1 (preparatory study) tested the LexMAL prototype to select the best items for the final LexMAL. The prototype was tested with two distinct groups of Malay speakers, namely Malay L1 (*N* = 60) and L2 (*N* = 60) speakers, to examine its ability to discriminate the two groups of Malay speakers based on their vocabulary size estimates. Furthermore, we followed LexTALE (Lemhofer & Broersma, 2012) and its extensions^2^ (e.g., Wen et al., 2023) to validate LexMAL with external criterion measures including self-rated proficiency, bidirectional translations, and a pre-existing language proficiency test that consists of multiple-choice questions. As far as we are aware, there is no freely available standardized Malay vocabulary test that can be used for the criterion comparison. Therefore, we used multiple-choice cloze questions from Malay sample examination papers as an alternative criterion measure in addition to Malay–English bidirectional translations and self-rated proficiency (following Wen et al., 2023). In addition, Mandarin–Malay translation tasks were presented to Malay L2 speakers^3^ to assess their Malay vocabulary knowledge in relation to their L1 (i.e., Mandarin Chinese, henceforth Mandarin).

The multiple-choice cloze test and translation tasks are complementary in that one assesses receptive word knowledge whereas the other assesses productive word knowledge. Each question of the multiple-choice cloze test contains a sentence with one word removed and this is incorporated into multiple-choice items, which require test-takers to select the appropriate option to fill in the blank. Bidirectional translations, on the other hand, are productive tasks in which test-takers are required to type in the target word form. These two measures have been used as criterion measures in previous studies and have been found to consistently correlate with receptive vocabulary size (Lemhöfer & Broersma, 2012; Nakata et al., 2020; Wen et al., 2023; Zhang et al., 2020).

The final version of LexMAL was constructed based on the results of Experiment 1. It consists of 60 words and 30 nonwords that cover a wide range of difficulty levels and show the greatest discriminatory power. In Experiment 1, sensitivity of the LexMAL prototype was examined by comparing LexMAL scores between the Malay L1 and L2 speakers, whereas its convergent validity was assessed by examining the correlations between LexMAL scores and participants’ performance in the translation and cloze tasks. The validity evidence of the final LexMAL was evaluated in Experiment 2 (validation study). We expected Malay L1 speakers to score higher than L2 speakers in LexMAL, reflecting the larger Malay vocabulary size expected in the L1 speakers. In addition, LexMAL was expected to show good internal reliability and good convergent validity and outperform self-ratings in predicting speakers’ translation and cloze test scores.

## Experiment 1: Preparatory study

### Method

#### Participants

An a priori power analysis conducted using G*Power (Faul et al., 2007) indicated that at least 51 participants were required for each language group to obtain .80 power to detect a medium effect size of .50 at the standard .05 alpha error probability. The present study recruited a slightly larger sample than recommended to account for unforeseen issues in online studies such as incomplete surveys or dropouts. Sixty Malay L1 speakers (13 males and 47 females) and 60 proficient Malay L2 speakers (all spoke Mandarin as L1; 13 males and 47 females) were involved in this study. The Malay L1 and L2 speakers were recruited based on their self-reported language background. All Malay L1 speakers identified Malay as their L1 and dominant language (except for one who identified English as their L1, exposed to Malay at the age of 9 and continued to use Malay as their dominant language). All Malay L2 speakers but four (who reported to have been exposed to Mandarin and Malay simultaneously during childhood) reported to have acquired their L1 (Mandarin) before Malay and use Mandarin as their dominant language. Their language background was verified using data from the language background questionnaire (see Task 5 in the stimuli section). Importantly, the average self-rated Malay language proficiency among the Malay L1 speakers was higher than the L2 speakers, *t*(118) = 10.60, *p* < .001 (see Table 1 for the summary of speakers’ language background).

**Table 1:** Summary of participants’ language background

Variable	Malay L1		Malay L2	
	Mean	*SD*	Mean	*SD*
Age (years)	23.92	3.21	25.82	4.75
Age of acquisition (years)				
Malay	0.50	1.54	5.05	1.69
English	4.60	2.25	4.28	2.09
Mandarin			0.57	1.63
Self-rated proficiency				
Malay	6.39	0.86	4.80	0.77
English	5.15	0.81	5.03	0.82
Mandarin			6.14	0.73

Language background questionnaire measured self-rated proficiency on a seven-point scale (1 = *very poor*, 7 = *native-like*).

All participants recruited were current students or graduates of tertiary education and had a minimum “Pass (C)” qualification for the *Bahasa Melayu* (Malay) and *Bahasa Inggeris* (English) subjects in the national high school examination (commonly known as the *Sijil Pelajaran Malaysia*, SPM). Participants received monetary compensation for their participation.

#### Stimuli

The present experiment involved five tasks to assess different Malay language skills and to collect self-rated language proficiency and language background information. Details of the stimuli used in each of these five tasks are described in the following subsections. Instructions were presented in English throughout the study, except for the instructions used in the LexMAL prototype, which were presented in Malay. The tasks and the items within each task were presented in the same order to all participants.

##### Task 1: LexMAL prototype

Ninety words were selected from the Malay–English translation norms (Lee et al., 2022). Following the recommendation of previous studies (Amenta et al., 2020; Brysbaert, 2013; Chan & Chang, 2018; Izura et al., 2014), the 90 words were selected from the full range of frequency bands to ensure that the test covered high frequency words that are most likely to be known by most speakers, as well as low frequency words that are more likely to be known only by highly proficient Malay dominant speakers. Table 2 summarizes the distribution of word stimuli across five frequency bands in Zipf values (van Heuven et al., 2014). From each frequency band, we sampled both easy (accuracy rate > 50%) and difficult word items (accuracy rate < 50%; based on the lexical decision accuracy data acquired from the Malay Lexicon Project, Yap et al., 2010). The final word list consisted of 46 nouns, 27 verbs, and 17 adjectives. Of these words, 60 were root words and 30 were words with circumfixes.

**Table 2:** Distribution of word stimuli across frequency bands (in Zipf values)

Frequency band	Total number of words	Words with Acc_LD_ > .5			Words with Acc_LD_ < .5		
		*n*	*M*	*SD*	*n*	*M*	*SD*
Zipf < 3.0	21	7	.70	.11	14	.27	.14
3.0 ≤ Zipf < 3.5	25	8	.65	.10	17	.28	.13
3.5 ≤ Zipf < 4.0	20	7	.69	.12	13	.35	.14
4.0 ≤ Zipf < 5.0	20	15	.78	.16	5	.22	.20
Zipf > 5.0	4	4	.95	.03	-	-	-

*Acc*_*LD*_ Lexical decision accuracy rate obtained from Yap et al. (2010).

In addition to the 90 words, 90 pronounceable nonwords were also included in the LexMAL prototype to correct for response bias (e.g., participants answering “yes” to every stimulus to increase their scores). These nonwords were generated based on another set of 90 source words selected from the Malay–English translation norms (Lee et al., 2022) using the same selection criteria as for the word stimuli. Word frequency of the source words (*M* = 3.62, *SD* = 0.63) were matched with the word stimuli in LexMAL prototype (*M* = 3.60, *SD* = 0.67), *t* = 0.27, *p* = .79, *d* = 0.04. A nonword generator, Pseudo (van Heuven, 2020) was employed to create nonwords (pseudowords) with legal letter combinations (bigrams and trigrams) in Malay. To achieve that, pseudo randomly substituted one letter of the source words and checks the legality of the letter combinations within the nonword using bigrams and trigrams extracted from a corpus of 34,326 Malay words from the Malay Lexicon Project (Yap et al., 2010) and open-source spell checkers (Aspell^4^ and Hunspell^5^). A set of 90 generated pseudowords were matched with the word stimuli in terms of word length (*M*_word_ = 7.39, *SD*_word_ = 2.69; *M*_pseudoword_ = 7.28, *SD*_pseudoword_ = 2.97; *t* = 0.26, *p* = .79, *d* = 0.04) and orthographic neighborhood size (*M*_word_ = 4.42, *SD*_word_ = 4.79; *M*_pseudoword_ = 4.64, *SD*_pseudoword_ = 4.61; *t* = 0.32, *p* = .75, *d* = 0.05). The 90 nonwords were also checked against two Malay dictionaries, *Kamus Melayu-Inggeris Dewan* (Jasmani, 2012) and *Kamus Perdana* (Cheng & Lai, 2019) to check that these nonwords are not real words in Malay. Finally, a LD1NN algorithm check (Keuleers & Brysbaert, 2011) was conducted on the combined list of words and pseudoword stimuli to verify that there was no inherent bias between the two stimuli sets, *z* = – 0.95, *p* = .34. Vocabulary knowledge is needed for test-takers to correctly identify words and pseudowords stimuli in the LexMAL prototype.

##### Task 2: Malay–English bidirectional translations

The Malay–English translation task consisted of 30 Malay nouns selected from Malay–English translation norms (Lee et al., 2022). To avoid ceiling performance of Malay L1 speakers, translation stimuli with a moderate to high level of difficulty were chosen. The selection of word stimuli followed the criteria set out in Lemhöfer and Broersma’s (2012) study, such that Malay (source) words with at least 50% translation error rates (including both omission and incorrect translations) and less than three possible English (target) translations were selected. The selected words were Malay nouns that could be translated into single-word English nouns. These criteria ensured that the Malay nouns selected for the task had a high difficulty level but were not too translation ambiguous. No cognates or words from the LexMAL prototype were included in the stimuli. In total, 21 root words and nine circumfixed words were selected, with a mean error rate of 70.00% (*SD* = 14.35), a mean number of possible translations of 1.83 (*SD* = 0.82), and a mean word frequency (Zipf value) of 3.67 (*SD* = 0.56).

Thirty English words were included in the English–Malay translation task. In total, 15 English words were taken from English–Malay translation norms (Lee et al., 2022) and a further 15 words with a similar translation difficulty were selected from English–Chinese translation norms (Wen & van Heuven, 2017a). Words from English–Chinese translation norms were included because we ran out of potential translation stimuli with similar difficulty in the Malay–English translation norms. Overall, the stimuli from the English–Malay translation norms had a mean error rate of 73.81% (*SD* = 16.51), a mean number of possible translations of 1.53 (*SD* = 0.83), and a mean word frequency (Zipf value) of 3.68 (*SD* = 0.55). The 15 English words from Wen and van Heuven (2017a) had a mean error rate of 62.44% (*SD* = 13.00), a mean number of possible translations of 1.93 (*SD* = 0.70), and a mean word frequency (Zipf value) of 3.33 (*SD* = 0.67). There was no significant difference between word frequencies (Zipf values) of words from both translation norms, *t* = 1.52, *p* = .14, *d* = 0.56.

To further check that there were no issues with the difficulty level in the translation tasks, a pilot study was conducted with ten Malay L1 and ten Malay L2 speakers. All items were translated correctly by at least one Malay L1 speaker. Neither floor nor ceiling effects were observed in the translation accuracy of the L1 (*M* = 51.50, *SD* = 11.80) and L2 (*M* = 32.67, *SD* = 12.25) speakers. The final complete set of stimuli is presented in Appendix C.

##### Task 3: Malay–mandarin bidirectional translations

A total of 30 Malay words were included in this task. Because there are no norms for Malay–Mandarin translation, 15 of the Malay words were selected from the Malay–English translation norms (Lee et al., 2022) and 15 words were selected from the English words of the English–Chinese translation norms (Wen & van Heuven, 2017a). Similar to the English–Malay translation task, words from English–Chinese translation norms were included to supplement the translation stimuli from Malay–English translation norms with similar translation difficulty. These English words were replaced with their Malay translation obtained from the *Kamus Dwibahasa* (Ibrahim, 2002) and the Oxford English-English–Malay Dictionary (Oxford University Press & Oxford Fajar, 2018). When an English word had more than one possible Malay translations, the Malay word that, according to *Kamus Perdana* (Cheng & Lai, 2019), had its dominant meaning matched with the dominant Mandarin translation (Wen & van Heuven, 2017a) was selected. No cognates were included, and all words were nouns. The word frequency (Zipf value) for the Malay stimuli from Malay–English (*M* = 3.66, *SD* = 0.53) and English–Chinese translation norms (*M* = 3.69, *SD* = 0.62) were matched, *p* = .88.

The Mandarin stimuli for the Mandarin–Malay translation task consisted of Mandarin translations of the 15 Malay words selected from the Malay–English translation norms (Lee et al., 2022), and 15 Mandarin dominant translations from the English–Chinese translation norms (Wen & van Heuven, 2017a). For Malay words that had more than one possible Mandarin translation, Mandarin words were chosen that had the dominant meaning of the Malay source words, according to Kamus Perdana (Cheng & Lai, 2019), and that matched with the English-dominant translations (Lee et al., 2022). Word frequency information for these Mandarin translations were obtained from Cai and Brysbaert (2010). Overall, the word frequency (Zipf values) for stimuli from the Malay–English (*M* = 3.85, *SD* = 0.76) and English–Chinese translation norms (*M* = 4.03, *SD* = 0.65) were matched, *p* = .50.

The translation stimuli were piloted using the same group of Malay L2 speakers that participated in the pilot for the stimuli of Task 2. No floor or ceiling effects were found (*M* = 46.17, *SD* = 14.64). However, two Mandarin (i.e., *炽热*/*bahang* and *心算*/*congak*) and three Malay items (i.e., *tikai*/*差别*, *komplot*/*阴谋* and *istilah*/*术语*) from Mandarin–Malay and Malay–Mandarin translations respectively received no correct translation. As a result, these words were replaced with other words that matched the selection criteria mentioned above. The final set of words for this task is presented in Appendix C.

##### Task 4: Malay cloze task

**Fig. 1:**
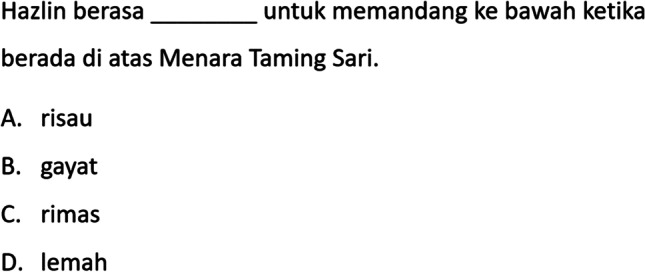
Example of cloze question

Cloze task is commonly used in vocabulary research to assess knowledge of collocations, and this measure correlates strongly with vocabulary size (González-Fernández, 2022; González-Fernández & Schmitt, 2020). The cloze task was used as an additional external criterion measure to validate LexMAL because there was no freely available standardized language proficiency measure for Malay. Twenty Malay cloze questions were selected from Malay sample examination papers that were designed for students of different education levels. Five easy questions (25%) were sampled from the *Ujian Pencapaian Sekolah Rendah* paper (UPSR - the official examination taken by Malaysian students at primary sixth grade). The other 15 questions (75%) were taken from the *Penilaian Tingkatan 3* (PT3 - the examination taken by Malaysian students at secondary third-form grade). The cloze questions involved a multiple-choice format (see Fig. 1 for an example).

The difficulty level of the cloze questions was piloted using six Malay L1 and seven Malay L2 speakers. As expected, the L1 speakers displayed high accuracy with smaller variation (*M* = 90.83, *SD* = 6.72), whereas the L2 speakers scored lower with higher variability (*M* = 58.57, *SD* = 15.29).

##### Task 5: Self-ratings and language background questionnaire

A language background questionnaire was created based on the Language History Questionnaire 3 (Li et al., 2019). The questionnaire was used to acquire information about participants’ multilingual language history and experience, such as participants’ age of acquisition, education history, and years and context of learning experience for all the known languages. The questionnaire also asked for self-rated proficiency for Malay, English, and Mandarin (Mandarin L1 participants only), using a scale from 1 (*very poor*) to 7 (*native-like*).

#### General procedure

The present experiment was administered online using Qualtrics (https://www.qualtrics.com). Participants were instructed to complete all tasks without external aids (e.g., dictionary). The study was approved by the Ethics Committee in the School of Psychology at the University of Nottingham Malaysia. Written consent was obtained from participants before data collection started.

The study started with the LexMAL prototype. Participants were required to make unspeeded yes/no decision to every stimulus presented to them, one at a time. The words and nonwords were presented to all participants in the same randomized order. Care was taken to ensure that in the random order stimuli of the same type (i.e., word/nonword) did not appear in four consecutive trials. Participants were required to indicate “yes” if they thought the letter string presented on the screen was an existing Malay word. They were told to respond “yes” to the stimulus even if they did not know the exact meaning of the letter string, but were certain that it was an existing Malay word. In cases where they thought the letter string was not a Malay word, or they were in doubt, they were instructed to respond “no”. They were also reminded that errors were penalized to control for response bias.

Next, participants completed the Malay–English translation task before the English–Malay translation task. Translation stimuli appeared one at a time on screen, and participants were required to enter the first translation that came to their mind. They could skip an item by indicating that they did not know the word or if they could not provide a translation. The Malay L2 speakers were presented with the Malay–Mandarin bidirectional translation tasks after completing the Malay–English bidirectional translations.

The Malay cloze task was presented after the translation tasks. Questions appeared on screen one at a time, and participants were required to select one correct answer out of four available choices. After that, the language background questionnaire was presented as the last part of the study.

### Results

Data of three participants from the L2 group were excluded from data analysis because response times in the LexMAL prototype of two participants was unusually fast (less than 300 ms for more than 5% of the trials), and the accuracy rate of a third participant was exceptionally low (18.33%).

Item assessment was conducted with the remaining data to examine the quality of all 90 word and 90 nonword items tested in the LexMAL prototype. The first subsection below reports the results of the item assessment and describes the process of item selection for the final version of LexMAL. Subsequently, the validity of the final LexMAL was evaluated by independent *t* tests to compare LexMAL scores between the two language groups. Additionally, convergent validity of LexMAL was examined via its correlations with the scores of other language tasks. The test reliability was computed using Cronbach’s alpha.

#### Item assessment

The approach used for the item assessment and selection of the final set of items for LexMAL was based on Wen et al. (2023). Behavioral data of the word and nonword items were assessed separately. Point-biserial correlations between the individual item responses and the overall test scores of participants were computed to assess predictiveness of each item to the overall test score. These correlations vary between – 1.0 and + 1.0. A positive point-biserial correlation indicates that good test performers (i.e., participants who obtained high overall scores) tend to identify the item correctly, when compared to weak test performers. In contrast, a negative point-biserial correlation reveals an atypical situation where the good test performers do less well on the item than the weak performers. Only items with positive point-biserial correlation were considered for the final version of LexMAL to achieve high test reliability (Izura et al., 2014).

Out of the 90 words, 86 had positive correlations and four words (i.e., “*ambak*”, “*juru*”, “*memijakkan*”, “*sementara*”) yielded negative correlations (*r*s < -.116). Likewise, all but two (88/90) nonwords showed positive correlations. The two nonwords that had negative correlations were “*surindam*” (*r* = – .126) and “*abi*” (*r* = – .243). The six items with negative correlations were removed from subsequent analyses.

**Fig. 2:**
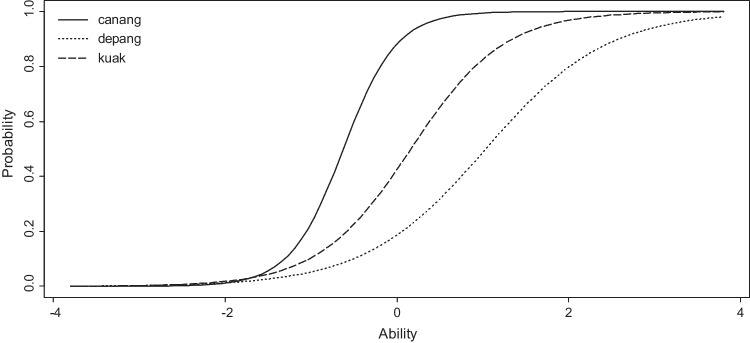
Example of item characteristic curves

Next, the items in the LexMAL prototype were assessed in terms of their discriminatory power. An item response theory (IRT) analysis was conducted to examine how well each test item distinguishes speakers according to their Malay proficiency (Amenta et al., 2020; Brysbaert, 2013; Izura et al., 2014; Salmela et al., 2021; Wen et al., 2023; Zhou & Li, 2022). Assuming that the items estimate vocabulary size, IRT analysis provides a measure of the difficulty level and the discrimination power of each item. For this purpose, a two-parameter logistic model in the *ltm* R package (Rizopoulos, 2006) was used to assess word and nonword items separately. The IRT analysis represents the speakers’ ability range on the *x*-axis, and the probability to answer the item correctly on the *y*-axis. The difficulty level of an item was operationalized by the ability level of participants who have 50% chance to answer the item correctly (i.e., at 0.5 probability). On the other hand, discrimination power, or how well an item can differentiate between speakers of different proficiency levels, was operationalized by the steepness of item response curve. The final set of the test items were chosen so that they span over the entire difficulty range and have steep item response curves. Figure 2 presents the item characteristic curves for three word items of LexMAL. Based on the curves, “*depang*” was more difficult than “*canang*” and “*kuak*”, whereas “*canang*” had higher discrimination power compared to the other two words.

The IRT analysis revealed three word items (“*mengehadkan*”, “*pemilihan*”, “*serta*”) with negative discrimination power, indicating that these items did not accurately discriminate between participants with high and low proficiency. Specifically, “*pemilihan*” and “*serta*” were rather easy words, hence all participants were able to identify the words. In contrast, “*mengehadkan*” was more consistently identified by participants with lower test scores, and missed by seven participants from the mid-to-high performance range. These three words (“*mengehadkan*”, “*pemilihan*”, “*serta*”) were excluded from the stimulus set. Subsequently, the remaining 83 words were ordered according to their difficulty level, from the lowest to the highest. Thirty difficulty groups were formed by grouping the ordered items into 23 groups of three items and seven groups of two items. Word items for the final LexMAL were selected by choosing two words with the highest discrimination power from each difficulty group (Amenta et al., 2020; Brysbaert, 2013; Izura et al., 2014; Salmela et al., 2021; Wen et al., 2023; Zhou & Li, 2022).

The IRT analysis of the nonwords revealed that all nonwords yielded discrimination power in the expected direction. Similar to the procedure used for the words, the 88 nonwords were ordered from the lowest to the highest difficulty level, and divided into 30 groups, in which 28 groups had three items and two groups had two items. The item with the highest discrimination power was selected from each difficulty group to form the final set of items for LexMAL. The above item selection procedure resulted in the most discriminative 60 word and 30 nonword items from the full range of difficulty levels. These final 90 items were selected for the final version of LexMAL. Table 3 summarizes the lexical information of the selected items.

**Table 3:** Lexical information of the final set of 60 words and 30 nonwords in LexMAL

Variable	Words		Nonwords	
	Mean	*SD*	Mean	*SD*
Number of letters	7.28	2.51	7.43	2.99
Orthographic neighborhood	4.62	4.87	3.87	4.14
Word frequency (Zipf)	3.56	0.54		

Orthographic neighborhood reported was Coltheart’s N (Coltheart et al., 1977). It was computed using the *vwr* R package (Keuleers, 2011).

#### Discriminatory power of different language tasks

The original LexTALE score ranges between 50 and 100% (Brysbaert, 2013). An alternative score was proposed by Brysbaert (the Ghent score). However, as pointed out by Wen et al. (2023), the Ghent score range depends on the number of words and nonwords included in the test. To enable comparison between different lextale inspired tests, Wen et al. (2023) proposed the use of normalized Ghent score (see equation shown below). It sums up the number of correctly identified words and penalizes the score base on guessing by the participant (“yes” responses for nonwords, i.e., false alarms). Normalized Ghent score ranges from – 100% to 100%, with a negative score indicating a higher false-alarm rate than correct word identification. This normalized Ghent score computation was also used for LexMAL.

$$\textrm{Normalized}\ \textrm{Ghent}\ \textrm{score}=\left({N}_{yes\ to\ words}-2{N}_{yes\ to\ nonwords}\right)\times \frac{100}{60}$$

For the scoring of the responses in translation tasks, the Malay–English translations provided by the participants were checked against four Malay–English dictionaries: *Kamus Melayu-Inggeris Dewan* (Jasmani, 2012), *Kamus Perdana* (Cheng & Lai, 2019), *Kamus Dwibahasa* (Ibrahim, 2002), and the Oxford English-English–Malay Dictionary (Oxford University Press & Oxford Fajar, 2018). Likewise, the Malay–Mandarin translations were checked against four Malay–Mandarin dictionaries, namely *Kamus Perdana* (Cheng & Lai, 2019), Kamus Kembangan (Lai, 2018), *Kamus Cina-Melayu Dewan* (Jasmani, 2013), and the Chinese Malay English Dictionary (Chinese-Malay-English Dictionary, 2019). Correct translations with grammatical affixation that do not change the meaning of root words, such as the use of third person singular ‘-s’ and plural ‘-s’ in English, were collated to its root word and accepted as correct responses. Words with affixations that have a different word meaning or word class than the correct translations were classified as incorrect responses. Translations with spelling errors were classified as correct when errors did not result in another word in the same language.

**Fig. 3:**
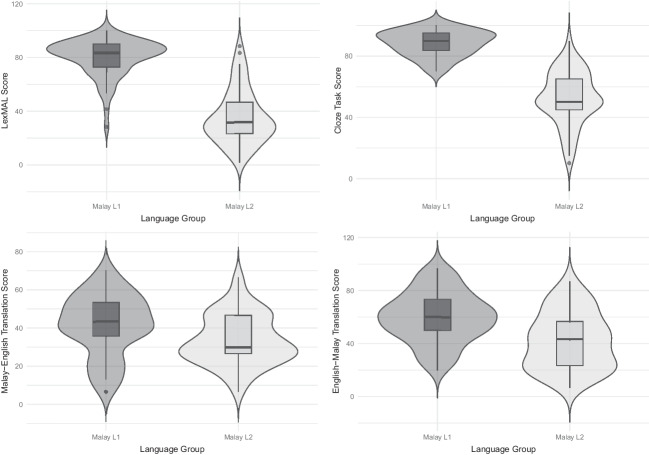
Distribution of Malay L1 and L2 speakers’ test scores for all language tasks

Independent *t*-tests were conducted to compare the performance between Malay L1 and L2 participants (see Table 4 for the average scores of each language group). As predicted, the Malay L1 group outperformed the Malay L2 group in all language tasks. Figure 3 summarizes the distribution of the performance gap between the L1 and L2 participants for each language task. Specifically, the L1-L2 differences were larger for LexMAL and cloze test compared to that of translation tasks.

**Table 4:** Test scores of all language tasks for both language groups

Language Tasks	Malay L1 (*n* = 60)		Malay L2 (*n* = 57)		*t*	*df*	Cohen’s *d*
	Mean	*SD*	Mean	*SD*			
LexMAL	90.04	6.88	67.75	10.04	13.95**	98.49	2.59
Malay–English bidirectional translation							
Malay–English	41.61	16.08	33.80	13.08	2.87*	115	0.53
English–Malay	59.83	18.97	41.93	21.33	4.80**	115	0.89
Combined	50.72	16.63	37.87	16.27	4.22**	115	0.78
Malay cloze test	88.33	8.32	52.63	17.35	14.08**	79.54	2.62

* *p* ≤ .05; ** *p* < .001.

#### Correlations of LexMAL with other language tasks

**Fig. 4:**
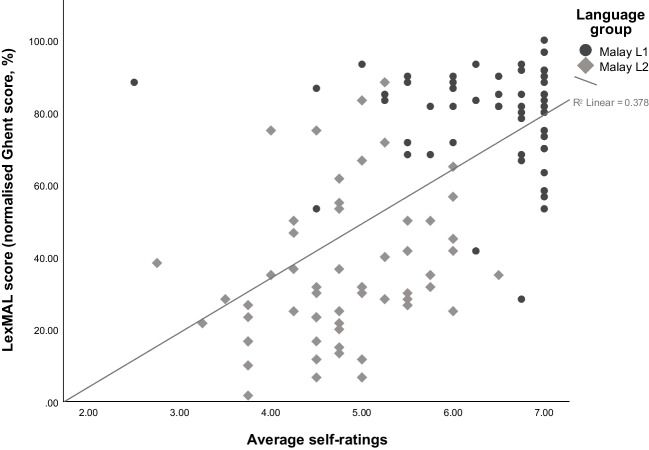
**C**orrelation between self-ratings and LexMAL scores

Correlational analyses were conducted to investigate the relationship between LexMAL and self-rated Malay proficiency with other vocabulary knowledge measures. Table 5 summarizes the Pearson’s correlation coefficients. LexMAL scores and self-ratings of all participants correlated positively and moderately with scores of the other language tasks. Importantly, LexMAL normalised Ghent score and self-rated proficiency were strongly correlated. It is important to point out that participants with identical self-rated proficiency varied considerably in terms of their LexMAL score (e.g., 95% *CI* [49.04, 81.29] at self-rated proficiency of 6 – *very good*, as demonstrated in Fig. 4). Furthermore, LexMAL scores discriminated better between Malay L1 and L2 speakers, because Malay L1 speakers (e.g., 95% *CI* [74.48, 92.85] at self-rated proficiency of 6 – *very good*) systematically scored higher than L2 speakers (e.g., 95% *CI* [27.70, 65.63] at self-rated proficiency of 6 – *very good*) even when they rated their Malay proficiency at the same level.

**Table 5:** Correlations of LexMAL scores and self-ratings with other language tasks

Predictor	All participants (*N* = 117)				Malay L1 (*n* = 60)				Malay L2 (*n* = 57)					
	Lex	ME	EM	Malay cloze	Lex	ME	EM	Malay cloze	Lex	ME	EM	MC	CM	Malay cloze
LexMAL	1.00	.37***	.51***	.78***	1.00	.18	.20	.37**	1.00	.40**	.41**	.62***	.34*	.42***
SR														
Listening	.52***	.32***	.43***	.63***	– .08	.21	.11	– .03	.13	.22	.35*	.34*	.44***	.40**
Speaking	.63***	.31***	.41***	.64***	.04	.15	.08	– .05	.34*	.24	.34*	.44***	.41**	.43***
Reading	.55***	.21*	.33***	.59***	– .09	.07	– .04	– .07	.20	.04	.22	.28*	.30*	.28*
Writing	.57***	.26**	.37***	.59***	– .01	.10	.07	.02	.25	.16	.23	.34*	.35*	.25
Average	.62***	.30***	.42***	.66***	– .04	.15	.06	– .03	.28*	.20	.34*	.42***	.45***	.41**

*SR* Self-ratings, *Lex* LexMAL, *ME* Malay–English translation, *EM* English–Malay translation, *MC* Malay–Mandarin translation, *CM* Mandarin–Malay translation.

The highest significant correlation in each column is bolded. * *p* < .05; ** *p* < .01, *** *p* ≤ .001.

To examine whether LexMAL scores outperformed self-ratings in terms of their correlation with other language tasks, Williams' (1959) *t* tests were conducted to compare the correlation strengths using the SPSS code from Weaver and Wuensch (2013). Results indicated that LexMAL scores correlated better than average self-ratings with Malay cloze test scores, *t*(114) = – 2.54, *p* = .01. No significant difference was found between LexMAL scores and average self-ratings for the correlations with Malay–English bidirectional translations, *t*s ≤ 1.28, *p*s ≥ .21. Furthermore, the correlation of LexMAL scores with Malay cloze test scores was significantly higher than its correlation with Malay–English translation, *t*(114) = – 5.65, *p* < .001, and English–Malay translation scores, *t*(114) = – 4.28, *p* < .001.

Interestingly, when the correlational analyses were restricted to the Malay L1 group, self-ratings and LexMAL no longer correlated with translation accuracy, *p*s ≥ .13, but LexMAL scores still correlated significantly with Malay cloze test scores, *r*(58) = .37, *p* = .003.

For Malay L2 speakers, LexMAL scores correlated positively with all other language tasks, *r*s ≥ .34, *p*s < .05. Similarly, their average self-ratings also correlated positively with all other language tasks, *r*s ≥ .34, *p*s < .05, except for their Malay–English translation scores, *p* = .14. With respect to correlation strength, Williams' (1959) *t* test indicated no significant difference between the correlations of LexMAL scores and average self-ratings with other language tasks, *t*s ≤ 1.58, *p*s ≥ .12. In other words, the correlation strength of LexMAL with (a) English–Malay translation; (b) Malay–Mandarin bidirectional translations; and (c) Malay cloze test scores were comparable to those of average self-ratings.

#### Reliability

Cronbach’s alpha returned a high reliability score for the final LexMAL at .94, .82 when the analysis was restricted to the Malay L1 group and .84 when the analysis was limited to the Malay L2 group.

### Discussion

The 180-item LexMAL prototype was tested in Experiment 1 to select a final set of 90 items that span across a wide range of difficulty levels and that have the highest discriminative power. In addition to self-ratings (cf. Amenta et al., 2020; Brysbaert, 2013; Izura et al., 2014; Salmela et al., 2021; Zhou & Li, 2022), bidirectional translation tasks and a cloze test were used as the external criterion measure to validate LexMAL (cf. Lemhöfer & Broersma, 2012; Wen et al., 2023).

As predicted, the Malay L1 speakers outperformed the L2 speakers on all language tasks. Specifically, the largest effect sizes were found for LexMAL and the cloze test, indicating that these two tests are the most sensitive at detecting L1-L2 proficiency differences. Furthermore, LexMAL scores were positively correlated with translation and cloze test accuracies, providing evidence to support the validity of LexMAL as a Malay proficiency measure. In addition, the correlation between LexMAL scores and cloze test accuracy was significantly higher than that of self-ratings and cloze test accuracy, suggesting that LexMAL as an objective language measure provides a better Malay proficiency estimate for bilingual speakers. Overall, the validity evidence of LexMAL is in-line with LexTALE (Lemhöfer & Broersma, 2012) and its extensions (Amenta et al., 2020; Brysbaert, 2013; Chan & Chang, 2018; Izura et al., 2014; Salmela et al., 2021; Wen et al., 2023; Zhou & Li, 2022).

Interestingly, LexMAL scores and self-rated proficiency did not correlate with translation accuracy when the analysis focused only on the L1 group. The larger variation that L1 speakers displayed in the translation tasks might be the reason for the lack of a significant correlation. Because the recall of word forms (as required by the translation task) is more difficult than word recognition (as required by LexMAL), L1 speakers showed more variance in translation tasks compared to LexMAL and self-rated proficiency (see Table 4). However, it is important to note that LexMAL is fundamentally a receptive vocabulary test, and as such, its scores are expected to correlate stronger with the receptive criterion measure (i.e., cloze test accuracy). Because LexMAL scores consistently correlated with cloze test scores across language groups, the convergent validity of LexMAL as a receptive vocabulary measure for both L1 and L2 speakers is well supported by our findings.

## Experiment 2: Validation study

Experiment 1 demonstrated the validity and reliability of LexMAL as a vocabulary size measure for Malay L1 and L2 speakers. Participants in Experiment 1 were presented with the 180-item LexMAL prototype. Because the items in the final LexMAL test were reduced to 90, it is important to replicate the reliability and validity of LexMAL. Thus, the 90-item final LexMAL was tested with another group of Malay L1 and L2 speakers.

### Methods

#### Participants

The same recruitment criteria and general procedures from Experiment 1 were followed for this validation study. A total of 122 Malay L1 (*N* = 61, 15 males and 46 females) and L2 speakers (*N* = 61, 15 males and 46 females) were recruited. The Malay L1 and L2 speakers were grouped using the same criteria as in Experiment 1 (see Participant section of Experiment 1). All but one Malay L1 speaker identified Malay as their L1 and dominant language (they acquired English as their L1 before the acquisition of Malay at the age of five, which later also became their dominant language). All Malay L2 speakers acquired their L1 (Mandarin) before Malay, except three participants who reported simultaneous exposure to Mandarin and Malay since birth. Importantly, these three participants identified Mandarin as their dominant language, just as other participants from the same language group. Similar to Experiment 1, the Malay L1 speakers’ self-ratings for Malay proficiency were significantly higher than the L2 speakers, *t*(119.73) = 12.11, *p* < .001 (see Table 6 for speaker’s language background summary).

**Table 6:** Summary of participants’ language background

Variable	Malay L1		Malay L2	
	Mean	*SD*	Mean	*SD*
Age (years)	23.15	4.21	25.70	4.81
Age of acquisition (years)				
Malay	0.13	0.67	4.51	1.63
English	4.15	2.64	4.28	2.13
Mandarin			0.51	1.06
Self-rated proficiency				
Malay	6.25	0.76	4.54	0.80
English	5.38	0.65	4.74	0.73
Mandarin			5.90	0.87

Language background questionnaire measured self-rated proficiency on a seven-point scale (1 = *very poor*, 7 = *native-like*).

#### Stimuli and procedure

The final 90-item LexMAL was used in Experiment 2. Other tasks included in Experiment 2 (translations, cloze task, and questionnaire) were identical to those used in Experiment 1. The procedure was identical to Experiment 1. The study was approved by the Ethics Committee of the School of Psychology at the University of Nottingham Malaysia. All participants provided informed consent at the beginning of the study.

### Results

To evaluate validity of the 90-item final LexMAL, independent *t* tests were conducted to compare LexMAL scores between the two language groups. Additionally, correlational analyses were conducted to evaluate convergent validity of the final LexMAL with the scores of other language tasks. The test reliability was computed using Cronbach’s alpha.

#### Discriminatory power of different language tasks

Table 7 summarizes the average scores of participants across different language tasks. Overall, the participants’ performance was comparable to that of Experiment 1, except that the Malay L1 speakers’ mean LexMAL score was significantly lower than that of L1 speakers in Experiment 1, *t*(109.66) = 2.39, *p* = .02, *d* = 0.43. Similar to Experiment 1, there was a significant difference between the LexMAL scores of the L1 and L2 groups, with a large effect size.

**Table 7:** Test scores of all language tasks for both language groups in Experiment 2

Language tasks	Malay L1 (*n* = 61)		Malay L2 (*n* = 61)		*t*	*df*	Cohen’s *d*
	Mean	*SD*	Mean	*SD*			
LexMAL	86.46	9.45	67.42	10.39	10.59**	120	1.92
Malay–English bidirectional translation							
Malay–English	40.82	11.92	36.23	11.67	2.15*	120	0.39
English–Malay	55.85	14.05	44.43	15.95	4.20**	120	0.76
Combined	48.33	10.18	40.33	10.86	4.20**	120	0.76
Malay cloze test	86.48	8.77	51.23	17.55	14.03**	88.22	2.54

* *p* ≤ .05; ** *p* < .001.

#### Correlations of LexMAL with other language tasks

LexMAL scores correlated positively with the scores of all other language tasks and self-ratings, hence replicating the convergent validity of LexMAL in Experiment 1 (see Table 8). In addition, as in Experiment 1, Williams’ (1959) *t* test was conducted to compare the correlation strengths of LexMAL scores and self-ratings with other language tasks using the SPSS code from Weaver and Wuensch (2013). Results revealed that the correlation strength between LexMAL scores and cloze test scores was significantly higher than that of Malay–English translation, *t*(119) = 4.51, *p* < .001, and English–Malay translation, *t*(119) = 3.63, *p* < .001. There was no significant difference between the correlation strength of LexMAL scores and average self-ratings with all other language tasks, *t*s ≤ .78, *p*s ≥ .44.

**Table 8:** Correlations of LexMAL scores and self-ratings with other language tasks in Experiment 2

Predictor	All participants (*N* = 122)				Malay L1 (*n* = 61)				Malay L2 (*n* = 61)					
	Lex	ME	EM	Malay cloze	Lex	ME	EM	Malay cloze	Lex	ME	EM	MC	CM	Malay cloze
LexMAL	1.00	.28**	.39***	.69***	1.00	.15	.18	.41**	1.00	.25	.24	.31*	.34**	.29*
SR														
Listening	.63***	.26**	.35***	.69***	.18	.23	.10	.03	.28*	.14	.15	.12	.14	.35**
Speaking	.67***	.25**	.36***	.69***	.41**	.20	.19	.26*	.31*	.11	.15	.14	.22	.37**
Reading	.63***	.20*	.27**	.66***	.27*	.20	.04	.24	.32*	.01	.02	.33**	.02	.30*
Writing	.58***	.25**	.27**	.61***	.30*	.30*	.05	.40**	.28*	.02	.11	.19	.21	.23
Average	.68***	.26**	.34***	.72***	.36**	.28*	.12	.30*	.34**	.08	.12	.23	.17	.36**

*SR* Self-ratings, *Lex* LexMAL, *ME* Malay–English translation, *EM* English–Malay translation, *MC* Malay–Mandarin translation, *CM* Mandarin–Malay translation.

The highest significant correlation in each column is bolded. * *p* < .05; ** *p* < .01, *** *p* ≤ .001.

As in Experiment 1, when the analysis was restricted to the L1 group, LexMAL scores correlated positively with cloze test scores. Intriguingly, unlike Experiment 1, self-ratings of the L1 group, but not their LexMAL scores correlated positively with their Malay–English translation scores. The average self-ratings also correlated with cloze test scores. In terms of correlation strength, there was no significant difference between the correlation of LexMAL and self-ratings with cloze test scores, *t*s ≤ .84, *p*s ≥ .40.

For the Malay L2 group, LexMAL scores continued to correlate positively with Malay–Mandarin bidirectional translations and cloze test scores, whereas average self-ratings only correlated with the latter. With respect to correlation strength, Williams’ (1959) *t* test did not detect a significant difference between the correlation strengths of LexMAL scores and average self-ratings with cloze test scores, *t*s ≤ .45, *p*s ≥ .65.

Reliability analysis revealed that the Cronbach’s alpha for final LexMAL was .92. When the analysis was restricted to either Malay L1 or L2 group only, the Cronbach’s alpha remained high at .85.

#### Discriminatory ability of LexMAL

In clinical settings, a receiver operator characteristic (ROC) curve analysis is frequently used to assess how well a diagnostic test can differentiate between two groups (e.g., people with or without a disease; Lalkhen & McCluskey, 2008; Read et al., 2016). Using a ROC curve analysis, Wen et al. (2023) proposed an optimum cut-off score that can discriminate between Mandarin L1 and L2 speakers with high sensitivity and specificity. To determine if LexMAL can distinguish between Malay L1 and L2 speakers, a ROC curve was plotted using the *pROC* R package (Robin et al., 2021).

**Fig. 5:**
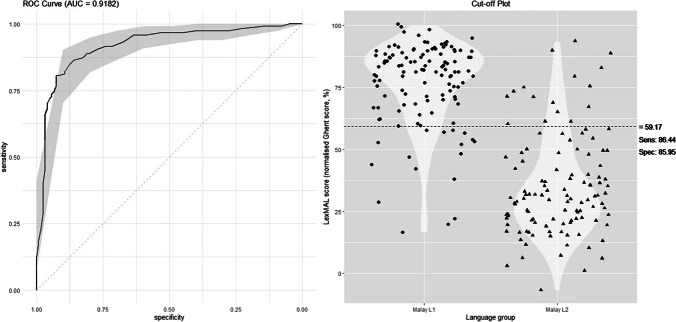
ROC curve with data from Experiment 1 and 2

Figure 5 presents the ROC curve for LexMAL plotted using data from both Experiment 1 and 2. LexMAL’s true positive rate (sensitivity) was plotted on the *y*-axis and false positive rate (1 – specificity) was plotted on the *x*-axis. The area under the ROC curve (AUC) measures LexMAL’s ability to discriminate between L1 and L2 speakers’ vocabulary scores, where an AUC of .5 indicates no discrimination ability, whereas an AUC of 1.0 indicates perfect discrimination (Hoo et al., 2017). The optimal cut-off point for LexMAL scores was also identified using point closest-to-(0, 1) corner method. The curve had an AUC of .918, suggesting that the proficiency of Malay L1 speakers, as indicated by LexMAL scores, correctly discriminated from L2 speakers 91.8% of the time. An optimal cut-off point for LexMAL scores was identified at 59.2%, with the sensitivity and specificity of LexMAL being 86.4 and 86.0%, respectively.

### Discussion

The final 90-item LexMAL was tested in Experiment 2 with a new group of participants. When Malay L1 and L2 speakers were compared in terms of their performance on all language tasks, it was found that L1 speakers consistently outperformed L2 speakers with large effect sizes (Cohen’s *d* > 0.39). Importantly, similar to Experiment 1, the largest effect sizes were found for LexMAL and cloze test accuracies. This finding suggests that discarding the stimuli with lower discrimination power from LexMAL prototype (in Experiment 1) does not reduce its discriminative sensitivity. LexMAL remains useful in discriminating the Malay proficiency of L1 and L2 speakers, and this is further supported by the ROC curve. Importantly, the ROC curve analysis indicated high sensitivity (LexMAL’s accuracy in identifying L1 speakers: 86.44%, see Fig. 5) and specificity (LexMAL’s accuracy in identifying L2 speakers: 85.95%, see Fig. 5) of LexMAL with a cut-off score of 59.17%.

In concordance with Experiment 1, the convergent validity of LexMAL is demonstrated by the positive correlations between LexMAL score and other language task accuracies. LexMAL scores predicted the bilinguals’ translation and cloze test performance. Furthermore, both LexMAL scores and self-rated proficiency correlated strongly with cloze test accuracy, with no significant difference observed between the two correlation strengths. In other words, LexMAL scores and self-rated proficiency predicted cloze test performance equally well in Experiment 2.

## General discussion

The present study was conducted to create a quick valid Malay yes/no unspeeded vocabulary test to measure the proficiency of Malay L1 and L2 speakers. Following the procedures to create a valid vocabulary test used for LexTALE (Lemhöfer & Broersma, 2012) and its extensions (Amenta et al., 2020; Brysbaert, 2013; Chan & Chang, 2018; Izura et al., 2014; Salmela et al., 2021; Wen et al., 2023; Zhou & Li; 2022), a LexMAL prototype was tested in Experiment 1. This prototype involved a large stimulus set (180 stimuli) that was tested with two groups of speakers in Experiment 1. The final 90-item LexMAL was selected based on the results of Experiment 1 and the final LexMAL was tested in Experiment 2.

Due to a lack of freely available objective language proficiency test in Malay, past research has resorted to estimating Malay proficiency using self-reported measures such as order of language acquisition (e.g., Lee & Low, 2014; Yap et al., 2017) or self-ratings (e.g., Jalil et al., 2011; Rahman et al., 2018; Rusli & Montgomery, 2020). However, considering most of the Malaysian Malay L2 speakers have a rather uniform age of Malay acquisition due to compulsory language education in school and their diverse language use and experience (Jin et al., 2013), individual differences in language proficiency of the bilingual or multilingual speakers can be difficult to assess based on just self-reported information. Hence, LexMAL as a freely available validated Malay proficiency test serves as a useful remedy that can objectively measure the proficiency of Malay L1 and L2 speakers for research purposes.

Just as LexTALE and its extensions, LexMAL is a yes/no unspeeded lexical decision task. Participants have to respond to one stimulus at a time by deciding yes or no depending on whether the letter string is a real word (Lemhöfer & Broersma, 2012). The validity of LexMAL was supported by the findings of both Experiments. Results showed that LexMAL scores can distinguish between Malay L1 and L2 speakers. Compared to other lextale extensions a similar effect size was found (see Table 9 for summary). Furthermore, no ceiling effect was observed for Malay L1 speakers and there was no floor effect with Malay L2 speakers. Thus, similar to Lextale_Fr (Brysbaert, 2013), Lextale_Esp (Izura et al., 2014), LEXTALE_CH (Chan & Chang, 2018), and LexCHI (Wen et al., in press), LexMAL can be used with L1 and L2 speakers.

**Table 9:** Comparisons of LexMAL scores with previous studies involving lextale extensions

Test	L1 speakers			L2 speakers			Cohen’s *d*
	*N*	Mean	*SD*	*N*	Mean	*SD*	
LexMAL	60	90.0	6.9	57	67.8	10.0	2.59
Lextale_Fr	152	76.4	12.0	164	14.8	20.7	3.64
Lextale_Esp	91	89.8	11.0	123	19.8	29.8	3.11
LEXTALE_CH	49	73.2	9.8	15	25.8	19.8	2.91
LexITA	58	96.6	3.6	141	34.0	-	-
Lexize	117	89.4	16.6	159	39.3	27.6	-
LextPT	130	91.5	6.8	120	49.1	23.2	2.52
LexCHI	54	91.7	13.2	75	43.6	29.0	-

All means are normalized Ghent scores (Wen et al., 2023).

Malay L1 speakers in this study consistently outperformed the L2 Malay speakers in all language tests. However, it is worth noting that the translation task was not as sensitive as LexMAL and cloze test in discriminating speakers based on their proficiency level (as can be seen in Fig. 3 there is large overlap in the translation scores of L1 and L2 speakers). The effect sizes of the performance differences were also smaller compared to LexMAL scores and cloze test scores (see Table 4). In terms of practicality, translation tasks are restricted to studies that involve bilinguals who speak the same language combination (e.g., English–Dutch, Lemhöfer & Broersma, 2012), and the scoring procedure is more time consuming compared to LexMAL and a cloze test. In summary, our findings indicate that LexMAL and cloze test are better options for studies seeking a quick and valid language proficiency measure of L1 and L2 speakers.

The convergent validity of LexMAL as a vocabulary measure was supported by significant correlations with translation and cloze test scores with moderate to large effect sizes (Cohen, 1988). In view of the high correlation between LexMAL scores and cloze test scores, one might easily assume that both LexMAL and cloze test are equally useful in measuring proficiency of Malay speakers. These two tests, however, are measuring different aspects of word knowledge. Specifically, a cloze test is a recognition test of collocations (knowledge of how words can be used together), whereas LexMAL is a test of form-meaning connections (i.e., vocabulary breadth). Correlations between these two tests were consistently found because knowledge of form-meaning connections to decode the meaning of words in sentences and word choices is necessary for correct responses to cloze questions (García & Cain, 2014; Gellert & Elbro, 2013; González-Fernández & Schmitt, 2020; Nation & Snowling, 1997; Schmitt, 2014). However, it is important to note that cloze tests adopt context-dependent testing, in which grammatical knowledge is also essential to comprehend the test items (Gellert & Elbro, 2013). In contrast, LexMAL presents words and nonwords in a de-contextualized manner (Amenta et al., 2020), which may provide a better estimate of construct distinct information about participants’ word knowledge (Read, 2000).

LexMAL scores were also strongly correlated with self-ratings, further supporting the validity of LexMAL as a language proficiency measure. Specifically, when all participants were taken into consideration (regardless of language group), participants who rated themselves with higher Malay proficiency tended to score higher on LexMAL. However, no significant correlation was found when the analysis was limited to the Malay L1 group. The correlation between self-ratings of L1 speakers with their vocabulary test scores varied within and across previous studies (Chan & Chang, 2018; Izura et al., 2014). In those studies, L1 speakers usually showed smaller variance in their high vocabulary test scores when compared to L2 speakers (see Table 9 for comparison). It is likely that the homogeneity of their L1 vocabulary size as a group was the explanation of the negligible–weak correlation observed between the vocabulary test scores and self-ratings (Brysbaert, 2013; Chan & Chang, 2018; Ferré & Brysbaert, 2017; Izura et al., 2014).

The subjectivity of self-ratings could also contribute to the lack of a correlation between the objective vocabulary measure and the subjective self-ratings of L1 speakers. Unlike the L2 speakers who had both their self-ratings and LexMAL scores spread across the proficiency range, the L1 speakers showed greater variability in their self-ratings than their LexMAL scores (see Fig. 4). When inspecting the LexMAL performance of Malay L1 and L2 speakers who gave themselves the same rating (e.g., 5/*good* – 6/*very good* in Fig. 4), the majority of the Malay L1 speakers appeared to score higher than the L2 speakers. This is possibly due to the difference in reference group used by the Malay L1 and L2 speakers when rating their language proficiency. Brysbaert (2013) reported that Lextale_Fr participants from the L1 group tended to be stricter in self-ratings because they compared their language ability to other highly proficient L1 speakers. In contrast, the L2 speakers were more lenient because they compared their proficiency to other relatively less proficient L2 speakers. Importantly, LexMAL scores, when compared to self-ratings, correlated better with cloze task performance. Taken together, these correlations replicated the findings of previous studies (Khare et al., 2013; Lemhöfer & Broersma, 2012; Tomoschuk et al., 2019; Wen & van Heuven, 2017a), indicating that objective measures like LexMAL are better estimates of language proficiency than subjective self-ratings.

Finally, the internal reliability analyses revealed that LexMAL is highly reliable in measuring the vocabulary size of Malay speakers. Due to the larger number of stimuli, it is not surprising that LexMAL’s reliability is higher than that of LexTALE (*a =* .81, Lemhöfer & Broersma, 2012). Such high reliability is also seen in other lextale extensions (Amenta et al., 2020; Brysbaert, 2013; Chan & Chang, 2018; Izura et al., 2014; Salmela et al., 2021; Wen et al., 2023; Zhou & Li, 2022).

The ROC curve of LexMAL also suggests that the LexMAL score is a very good classifier of Malay proficiency in terms of Malay L1 and Malay L2 speakers. Because Malay–English bilingual speakers in Malaysia use both languages in a variety of daily contexts from a very young age, it can be challenging for them to self-evaluate their L1 and L2 proficiencies and to indicate whether or not Malay is their L1. This is reflected in the less consistent prediction of self-rated proficiency on language task performance compared to LexMAL in the present study. Hence, in bilingual populations in which people use two languages frequently from an early age, or the age of acquisition or order of language acquisition does not necessarily reflect whether one or the other language is more proficient, an objective language proficiency measure like LexMAL provides a better estimate of language proficiency measure than self-ratings.

In addition, LexMAL can also be used as a screening test to decide if a Malay-speaking bilingual has the proficiency of a L1 or L2 speaker. However, it should be noted that LexMAL is designed to measure the proficiency of Malay L1 and L2 speakers using vocabulary knowledge as an estimate. Despite its usefulness in research that seeks practical and objective proficiency measure, the present study does not provide direct evidence for how the context independent LexMAL items measure written vocabulary knowledge (e.g., the vocabulary knowledge required for word recognition and recall). Therefore, future research is needed to pinpoint the extent to which the test measures the form-meaning knowledge by the moderately-highly proficient Malay speakers. With this restriction, researchers should be cautious when LexMAL scores from the present study are used as a reference.

## Conclusions

The present study described the development of LexMAL, a quick lexical test for estimating language proficiency in Malay. The validity and reliability of LexMAL as a Malay language proficiency measure was demonstrated, with no ceiling effect observed for the L1 speakers and no floor effect for L2 speakers. As far as we are aware, LexMAL is the first Malay lexical test that can reliably measure the proficiency of L1 and L2 speakers. LexMAL is useful for researchers in, for example, linguistics, psychology, and education that require a quick (less than 5 min), practical and objective proficiency measure. LexMAL can be taken online at https://www.lexmal.org/, or a paper and pencil version of LexMAL can be downloaded from https://osf.io/8y4ft/.

## Data Availability

The final LexMAL stimuli and instruction generated during the current study are available in the Open Science Framework repository, https://osf.io/8y4ft/.

## Appendix A: LexMAL instruction

*Salam sejahtera. Anda akan dikemukakan dengan sebuah ujian kosa kata Bahasa Melayu. Ujian ini mengandungi 180 percubaan, di mana rentetan huruf akan ditunjukkan pada setiap percubaan. Tugas anda adalah untuk menentukan sama ada rentetan huruf tersebut wujud sebagai perkataan Bahasa Melayu.*

*Tekan "Ya" sekiranya:*

- *Anda rasa rentetan huruf tersebut merupakan perkataan Bahasa Melayu yang sah, atau;*
- *Anda pasti perkataan tersebut wujud dalam Bahasa Melayu, tetapi tidak pasti maksud perkataan tersebut.*

*Sebaliknya, tekan “Tidak” kalau:*

- *Anda rasa rentetan huruf tersebut bukan perkataan Bahasa Melayu yang sah, atau;*
- *Anda tidak pasti sama ada rentetan huruf tersebut wujud sebagai perkataan Bahasa Melayu.*

*Anda dinasihatkan supaya tidak cuba meningkatkan markah anda dengan pilih “Ya” untuk “perkataan” yang tidak pernah anda temui. Hal ini demikian kerana setiap kesilapan akan ditolak markah. Anda boleh mengambil masa sepanjang yang diperlukan untuk setiap percubaan. Keputusan ujian ini hanya bermakna dengan syarat anda tidak menggunakan kamus dan menjawab ujian ini dengan usaha sendiri.*

## Appendix B: LexMAL stimuli

Note that W indicates word and N nonword

tempa (W), jijil (NW), garap (W), peres (W), berahi (W), persempadunan (NW), mengisahkan (W), kerawang (W), pelarasan (W), ruau (NW), genit (W), tagih (W), sanak (W), landai (W), engki (NW), meranapkan (W), tewai (NW), penataan (W), perikanan (W), sisuh (NW), pembentekan (NW), kelambu (W), amar (W), gerbang (W), pengimpunan (NW), penganatan (NW), mengotakan (W), depang (W), lincah (W), sasu (NW), memercikkan (W), unggap (NW), senyit (NW), perakuan (W), bentena (NW), mengasyikkan (W), perenggan (W), menjerniakan (NW), menggondakan (NW), cerca (W), buil (NW), pertumbulan (NW), pembiayaan (W), cambah (W), pemenjaraan (W), jati (W), kibal (NW), anjung (W), lekang (W), congik (NW), edar (W), menginsarkan (NW), mengadunkan (W), belantan (W), menyamankan (W), serakah (W), latap (NW), kelibat (W), perseteruan (W), persumbahan (NW), perkasa (W), tuding (W), sauh (W), peruncukan (NW), kuak (W), menjangkakan (W), lampat (NW), olak (W), hambar (W), timai (NW), canang (W), kayuh (W), meledakkan (W), persengketaan (W), bantuk (NW), seloroh (W), lafa (NW), menghalakan (W), tunjang (W), centap (NW), mutu (W), terjah (W), duka (W), palanu (NW), damar (W), salasilah (W), melujutkan (NW), ganyang (W), perhutanan (W), kendur (W)

## Appendix C: Translation stimuli

Malay–English translation stimuli (Malay, in bold), with the expected English translations.

***selendang***/shawl, ***perli***/sarcasm, ***citra***/image, ***salur***/channel, ***wakil***/representative, ***penyebutan***/pronunciation, ***perselisihan***/disagreement, ***penjajahan***/colonization, ***khianat***/treason, ***semboyan***/signal, ***muslihat***/trick, ***kenduri***/feast, ***percukaian***/taxation, ***susur***/exit, ***perkiraan***/calculation, ***penyelenggaraan***/maintenance, ***rencana***/article, ***benteng***/wall, ***nahas***/accident, ***budi***/kindness, ***peti***/box, ***perdagangan***/trade, ***wayang***/movie, ***judul***/title, ***khasiat***/nutrition, ***rangkap***/verse, ***sahut***/reply, ***peruntukan***/allocation, ***muara***/estuary, ***bidan***/midwife

English–Malay translation stimuli (English, in bold), with the expected Malay translations.

**tobacco**/*tembakau*, **slope**/*cerun*, **cemetery**/*perkuburan*, **mediation**/*pengantaraan*, **minister**/*menteri*, **agony**/*seksaan*, **expenditure**/*perbelanjaan*, **blanket**/*selimut*, **strip**/*jalur*, **specialization**/*pengkhususan*, **drought**/*kemarau*, **tassel**/*rumbai*, **conference**/*persidangan*, **salute**/*tabik*, **waist**/*pinggang*, **armament**/*persenjataan*, **bullet**/*peluru*, **foam**/*buih*, **pouch**/*dompet*, **journalism**/*kewartawanan*, **clown**/*badut*, **pearl**/*mutiara*, **ivory**/*gading*, **distinction**/*perbezaan*, **sarcasm**/*sindiran*, **schooling**/*persekolahan*, **craftmanship**/*pertukangan*, **wreckage**/*bangkai*, **stump**/*tunggul*, **cradle**/*buaian*

Malay–Mandarin translation stimuli (Malay, in bold), with the expected Mandarin translations.

***pembalasan*** */报答,* ***pemampasan****/补偿,* ***haluan****/方向,* ***maruah****/尊严,* ***perdamaian****/和平,* ***telapak/****脚板,* ***kebuluran****/饥荒,* ***ikrar****/誓言,* ***pengiktirafan****/承认,* ***tembaga****/铜,* ***pembaharuan****/改革,* ***simpang****/分歧,* ***kecekapan****/能力,* ***kanji****/淀粉,* ***obor****/火炬,* ***gempa****/震动,* ***penolakan****/推辞,* ***punca****/来源,* ***kenalan****/熟人,* ***saraf****/神经,* ***pengamatan****/监视,* ***saran****/建议,* ***bijian****/谷物,* ***laci****/抽屉,* ***bakti****/忠心,* ***pergaulan****/社交,* ***kedutan****/皱纹,* ***bahaya/****危险,* ***kubu****/堡垒,* ***lombong****/矿*

Mandarin-Malay translation stimuli (Mandarin, in bold), with the expected Malay translations.

***痛苦*** */kesengsaraan,* ***辩论****/bahas,* ***战斗****/pertempuran,* ***闪电****/kilat,* ***迫害****/penganiayaan,* ***湖****/danau,* ***灵感****/ilham,* ***顺序****/urutan,* ***内容****/isi,* ***嫩苗****/pucuk,* ***善行****/amal,* ***光辉****/semarak,* ***直觉****/naluri,* ***乞丐****/pengemis,* ***沙漠****/gurun,* ***附录****/lampiran,* ***习俗****/adat,* ***宝石****/permata,* ***下巴****/dagu,* ***烟囱****/serombong,* ***专门****/pengkhususan,* ***头衔****/gelar,* ***工资****/upah,* ***茅草****/lalang,* ***摩擦****/geseran,* ***拳头****/penumbuk,* ***尸体****/mayat,* ***同伴****/teman,* ***包装****/pembungkusan,* ***苔藓****/lumut*

## Footnotes

1 However, it is important to note that due to the differences in number of test items and difficulty level, LexMAL scores, like those of the other lextale extensions, cannot be directly compared to LexTALE scores as in the Dutch and German parallel versions.

2 Most of the lextale extensions were validated using self-rated proficiency only.

3 The Malaysian Chinese ethnic group makes up 24.6% of the Malaysian population and is the largest Malay L2 speaking ethnic group in Malaysia. They usually speak Mandarin as their L1 with some exceptions who speak other languages (e.g., English) as their L1.

4 https://ftp.gnu.org/gnu/aspell/dict/0index.html (accessed in December 2020)

5 https://github.com/titoBouzout/Dictionaries (accessed in December 2020)
